# Epidemic Puerperal Peritonitis

**Published:** 1850-06

**Authors:** Wm. Kenney

**Affiliations:** Millersburg, Ky.


### WESTERN JOURNAL

OF

### MEDICINE AND SURGERY.

JUNE, 1850.

### ART. I.—*Epidemic Puerperal Peritonitis.* Reported by WM. KENNEY, M. D., of Millersburg, Ky.

The panic of the epidemic of summer had passed—the public mind to tranquillity had been restored, and disease once more seemed to have passed away from our midst—autumn,

> "Crown'd with the sickle and the wheaten sheaf,
> ——————— nodding o'er the yellow plain,
> Comes jovial on,,'

when a respite from the arduous and responsible duties of the profession, from the calm succeeding the storm, we hoped was in store for us. But before the winter,

> ——————— "in sable cincture, shadows vast,
> Deep-tinged and damp,"

sets fairly in, the energies and benevolent purposes of the medical profession were again brought into requisition, by an epidemic, to administer unto the maternal portion of our community who had not, as yet, commenced the ordeal of a parturient female, or had scarcely finished the throes that nature in child-birth entails before they had visited upon them the prodromes of epidemic puerperal peritonitis, the declaration of which was soon in formidable array before us.

This truly Protean disease commenced its ravages in the latter part of November, continuing through December and January, carrying terror with it. But before entering fully into the consideration of the subject of our undertaking we will premise, and hope not without additional interest—a more detailed account of the disease by a reference to the epidemic of preceding years as has been noticed, in some of the works extant on diseases of females, from Hyppocrates down, (but to Dr. Denman, says a writer, the profession is indebted for the first distinct essay on Epidemic Puerperal Fever,) and in each year of its prevalence, though confined principally to populous cities, and the wards of lying-in hospitals, it has been marked by great fatality.

A gentleman writing about 1761, states that all women delivered by him in a lying-in hospital in London, died during the prevalence of the disease.

Dr. Leake, in his work on diseases of women, says: "That from December 13, 1768, to December 12, 1769, 180 women died. From December 12, 1769, to December 11, 1770, 270 women died. From December 11, 1770, to December 10, 1771, 172 women died."

Dr. Wm. Hunter says: "That of thirty-two patients who were attacked with the disease during two months, only one recovered. Various plans of treatment were put into execution—venesection, cooling medicines, cordials and stimuli."

Professor Young, of Edinburg, says: "In 1773, puerperal fever appeared in the lying-in ward of the Royal Infirmary, about the end of February, when almost every woman as soon, or within twenty-four hours after delivery was seized with it and died, though every method was used to cure the disorder."

Dr. Dewees, of Philadelphia, in his work on Diseases of Females, 1827, says: "This disease very rarely presents itself as an epidemic in this country, and the only record of the kind recollected by him was that of Dr. Jackson, when it prevailed in part of Pennsylvania, in the fall of 1817, and the spring of 1818, and though treated with both vigour and ability, about one half died."

Other and more or less favorable accounts have been given of the prevalence of this disease down to 1842, by various writers coincident with the prevalence of epidemic typhus fever, or erysipelatus inflammation, but no other forms of disease, save catarrhal affections common to the season, were present during the prevalence of that which forms the subject of this report, and the most of those attacked trace the commencement of their afflictions back to the prevalence of epidemic cholera in August and September, from which time they say they have not known a well day.

Though our field of observation was confined to that of a country village, where the cases were not so numerous as would have been during an epidemic of the kind in the wards of a lying-in hospital in a populous city, yet the loss was equally as severely felt, and the fatality as great in proportion. But nothing, however, occurred to support the opinion of the contagious character of the disease, contended for by some writers, farther than the fact mentioned by Dr. Condie, of Philadelphia, who says: "How otherwise can be explained the circumstances of the disease, in one district, being exclusively confined to the practice of a single physician, extensively engaged in obstetrical practice, while no instance of the disease has occurred in the patients under the care of any other accoucheur practising within the same district."

The majority attacked were of a delicate conformation with the scrofulous diathesis—more or less predominating, and varying from twenty to thirty-six years of age—occupying elevated positions in society, and blessed with an abundant means of comfort.

Abortions after the first month or six weeks of gestation, and premature labor, were occasionally to be met with during these months, and the majority of those attacked expressed themselves as not "looking" for some time yet, while others confined nearest the full term gave birth to children that to all appearance had been dead for days or weeks. Some again coming into the world, as supposed at maturity, presented for the first few days a promise to do well, but finally become jaundiced, and declined or wasted away as if with marasmus.

The labors in no instance, that we recollect, differed from ordinary labor of other seasons as to time, hemorrage or suffering.

The character of the disease that occured here, to a great extent, corresponded with that given to an epidemic, in some of the books as occurring in London, at Aberdeen, Leeds, Edinburg, and Dublin, with the addition of its adynamic or typhoid form. It appeared to effect primarily the peritoneal surfaces covering the uterus, and radiating from that point to other parts. The attack may be said, in some instances, to have taken place several days before, but in the majority of cases about the third or fourth day after confinement. The earliest symptoms denoting an abnormal condition of the system, predominating over that state of malaise the patient for some time had been accustomed to, were slight rigours, pain in the head and back, numbness of the extremities, acceleration of the pulse, flushing of the face, and increase of heat of surface, more or less abdominal pain and tenderness with internal heat and burning, diarrhea, with tormina and tenesmus.

Abdominal pain, heat, and burning, tormina, and tenesmus, diarrhea, or frequent and unavailing efforts at stool, with an occasional discharge of a little transparent mucous or muco-feculent matter, were the precursory symptoms of disease in those attacked before confinement.

The chill which rarely ever amounted to shivering, consisted rather of a sense of coldness on change of position, and was followed by flushing of one or both cheeks, increase of heat of surface and of thirst, burning of the palms of the hand and soles of the feet. The heat of surface soon subsided, followed by more or less partial perspiration, and rarely ever did it afterwards transcend the natural temperature of the body. The pulse attained one hundred and twenty beats per minute, small soft and compressible, generally, with a greater tendency to increased than diminished frequency.

Gastric irritability soon manifested itself as did bronchial irritation, with increased secretion of mucous of a semi-transparent or muco-purulent character; and in one instance the original disease was entirely lost sight of, and the patient carried off by ulceration of the mucous surfaces of the bronchia from deposition of tubercular matter, though the cough and bronchial secretion mostly, after a few days, became less harassing, and measurably subsided. The nausea and vomiting were truly obstinate and distressing, from the succussion it produced, and continued throughout the disease subject to occasional remissions and exacerbations. The matter vomited varied in character from a frothy saliva to transparent mucous, mucous of a tenacious and deep saffron-like color, and towards the termination in fatal cases the matter ejected became of a dark vitiated-like billiary matter.

Increased sensibility of the abdomen on pressure was early developed in the disease, confined at first to the hypogastric or iliac regions, and rather of a subacute and burning or aching character, as described by patients, that gradually extended itself over the abdomen, subject, occasionally, to modifications, but never at any time entirely subsiding. Nor do we recollect of the pain becoming sharp, except preceding an operation, or after a paroxysmal attack, nor was the abdomen so tender as to not permit of the weight of an ordinary amount of bed clothing, in addition to the fomenting cloths and bran poultices. Nor was there any great disposition on the part of the patient to keep the knees drawn up, but most generally the patient laid on the back, with the head slightly raised, with an inclination to slip down towards the foot of the bed, except late in the disease when, from restlessness, there would be desire for a change of position for a time, on either side.

The enlarged uterus, in the majority of cases, could be felt through the integuments above the symphisis pubis, and more than probably a dip of the inflammatory action, in some instances, may have taken place into the proper tissue of that organ, as was found to be the case, as is reported by **M. Duges** and **M. Lonnelle**, in patients examined by them.

A tumid and tympanitic state of the abdomen was one of the earliest symptoms recognised on being called to a patient. The abdomen presented a smooth polished surface, with the convolutions of the inflated bowels, in the thniner subjects, readily detected by the eye.

The lochia varied, in some it was entirely suppressed, in others for a time only. In some it underwent no change, while again you would find patients for the first few days, at intervals, as if by an expulsive effort of the uterus, discharging semi-coagulated masses of blood; and several of my patients expressed themselves as having felt an escape of gas from the womb. The vaginal mucous membrane was hot and congested, with frequently a more or less offensive muco-sanguineous discharge.

The secretion of milk was invariably suspended, and the mammary gland became perfectly flaccid, while the mother evinced no very great interest in the child.

The action of the heart, upon auscultation, emitted rather a feeble sound, and the pulse was one hundred and twenty strokes to the minute, increasing from one hundred and forty to one hundred and sixty, towards the close in fatal cases.

The tongue varied from a healthy appearance to a slight, thin, light colored coating, or a dark brown, dry in the center, and again you would find it with a red edge and tip, or of a deep, glossy, red color.

The thirst, after the first twenty-four or forty-eight hours, was not very remarkable, and drinks, when called for, were taken but sparingly.

Diarrhea was a constant accompaniment in the early part of the disease, and might be said, in many cases, throughout it, and in excess, yielding readily to no therapeutical means whatever. Relaxation of the sphincter ani, and involuntary discharges at an early period in the disease, were occasionally observed, and the diarrhea, instead of exerting a benign influence by its depletory action, as is supposed by some, resulted quite to the contrary. In some cases, after a time, the bowels yielded more kindly to the action of medicinal means, and the secretions acquired a more healthy character and consistence; coeval with which there was an improvement in the abdominal symptoms, in the state of the skin, and in the expression of the patient, without alteration in the force and frequency of the pulse. This condition formed a delusive truce, in the course of the disease, that was soon dissipated by the return of diarrhea, the precursor of renewed inflammation of a more acute character, acting, perhaps, on a more limited area, from the locality of the pain, and tenderness on pressure, which, at this stage of the patient's strength, lessened greatly the chances in their favor.

The dejections varied in consistence and character, presenting, in the commencement, something of a muco-feculent character, succeeded by a more copious discharge of a cider-like nature, with a filmy, curdled matter suspended in it, that was ultimately, in the last few days of life, followed by thin, watery evacuations of a dark-green or copperas-like color, extremely offensive, and attended with burning and scalding pain, giving an increased amount of tormina and tenesmus.

The urine, in concatenating the history of the morbid secretions of the system in this disease, was found diminished in quantity, and varied from a healthy to a turbid and deep ley-like color, with a cloud of mucus floating through it; and cases were not wanting in which there was a partial suppression and retention, that required, for days, the use of the catheter. Its acrid qualities, too, were equally manifest from the expression of the patient in micturition, during the course of the disease, and gave reason to believe it, in some instances, the cause of retention.

The heat of surface, as has been before noticed, was not very marked after the first day or two, and the perspiration that frequently followed, presented nothing abnormal as to smell, but seemed partial in its appearance, and as life began to show the doubtful tenure by which it was held, the extremities became cold. Petechia presented occasionally; and, in all of the fatal cases, sudamina were abundant on the upper part of the chest, both posteriorly and anteriorly.

The intellectual faculties, in one or two instances, became disordered early in the disease, and finally in all.—The delirium was of a low muttering kind, and strange fancies possessed some of them, so much so as to give it almost a monomaniacal character. The chiming of bells in the distance, the music of a choir, or a buzzing noise in the ears of patients were frequently a source of annoyance to them.

The contour of the face was very little altered, save by emaciation, and nothing of the "face grippe" which is spoken of in the books was noticed. The color was rather pale or sallow, with an occasional flush of one or both cheeks. Emaciation was more shown on the body and extremities than about the face. The expression of the eye rather vacant and unmeaning; a picking at the fingers and of the lips and nose, and a constant disposition was remarked, in the more protracted cases, to adjust the bed-clothing.

Sleep was short and disturbed, the appetite failed, and the irritability of the stomach increased, so as to reject the blandest fluids. There was hiccough, and a great disposition to syncope on assuming the erect position.

In nearly all cases, the disease was of a subacute character, or a tendency to that form of inflammation, and its duration was from days to weeks.

Occurring, as this disease did, in private practice, the possibility of pursuing its study by postmortem examination was precluded, but it was evident that there was peritoneal inflammation, thickening and more or less effusion, with inflammation of the peritoneal surfaces of other of the abdominal organs, occasionally, from the location and deeper-seated pain and tenderness.

About two-thirds confined were attacked, and under whatever therapeutical means were employed three-fourths of the cases, that presented themselves, terminated fatally.

It had never before been our misfortune to meet with an epidemic of the kind, and we find in this some difference from the usual symptomatology as regards the pulse, pain, delirium, position, diarrhea, the tolerance of remedies, acuteness of the patient's sufferings, tenderness, and the state of the secretions. The malignancy differed from that of sporadic puerperal peritonitis, and from what we learn from reports, of its greater extent of mortality.

The plan of treatment found most available, in this instance, consisted in fomentations; bran poultices; epispastics; free sponging with tepid water the body and extremities; mercurial frictions, as recommended by Armstrong, Vanduesand, and Velpeau; infusions of rhei and geranium maculatum, with laudanum; anodyne and astringent enemata; vaginal injections of warm milk and water; mucilaginous drinks; and stimulants, with attention to cleanliness, quietude, and diet.

Bleeding was but rarely resorted to, and then badly borne. Mercury alone, or combined with opiates in cathartic or alterative doses, rather aggravated the condition of the patient, by increasing the tympany and irritability of the bowels.

